# Carbohydrate-Active Enzymes in *Pythium* and Their Role in Plant Cell Wall and Storage Polysaccharide Degradation

**DOI:** 10.1371/journal.pone.0072572

**Published:** 2013-09-12

**Authors:** Marcelo M. Zerillo, Bishwo N. Adhikari, John P. Hamilton, C. Robin Buell, C. André Lévesque, Ned Tisserat

**Affiliations:** 1 Colorado State University, Department of Bioagricultural Sciences and Pest Management, Fort Collins, Colorado, United States of America; 2 Michigan State University, Department of Plant Biology, East Lansing, Michigan, United States of America; 3 Agriculture and Agri-Food Canada, Ottawa, Ontario, Canada; 4 Department of Biology, Carleton University, Ottawa, Ontario, Canada; Université Paris-Sud, France

## Abstract

Carbohydrate-active enzymes (CAZymes) are involved in the metabolism of glycoconjugates, oligosaccharides, and polysaccharides and, in the case of plant pathogens, in the degradation of the host cell wall and storage compounds. We performed an *in silico* analysis of CAZymes predicted from the genomes of seven *Pythium* species (*Py. aphanidermatum*, *Py. arrhenomanes*, *Py. irregulare*, *Py. iwayamai*, *Py. ultimum* var. *ultimum*, *Py. ultimum* var. *sporangiiferum* and *Py. vexans*) using the “CAZymes Analysis Toolkit” and “Database for Automated Carbohydrate-active Enzyme Annotation” and compared them to previously published oomycete genomes. Growth of *Pythium* spp. was assessed in a minimal medium containing selected carbon sources that are usually present in plants. The *in silico* analyses, coupled with our *in vitro* growth assays, suggest that most of the predicted CAZymes are involved in the metabolism of the oomycete cell wall with starch and sucrose serving as the main carbohydrate sources for growth of these plant pathogens. The genomes of *Pythium* spp. also encode pectinases and cellulases that facilitate degradation of the plant cell wall and are important in hyphal penetration; however, the species examined in this study lack the requisite genes for the complete saccharification of these carbohydrates for use as a carbon source. Genes encoding for xylan, xyloglucan, (galacto)(gluco)mannan and cutin degradation were absent or infrequent in *Pythium* spp.. Comparative analyses of predicted CAZymes in oomycetes indicated distinct evolutionary histories. Furthermore, CAZyme gene families among *Pythium* spp. were not uniformly distributed in the genomes, suggesting independent gene loss events, reflective of the polyphyletic relationships among some of the species.

## Introduction

Oomycetes (subphylum or class Oomycota) are part of the Stramenopiles and the supergroup Chromalveolates which likely originated from a biflagellate ancestor containing a red algal symbiont [1], [2]. It is hypothesized that the oomycetes lost their algal plastid over the course of evolution, and as a consequence, are non-photosynthetic organisms with an osmotrophic lifestyle and filamentous growth habit (mycelium), similar to true Fungi [3], [4]. However, unlike Fungi, oomycetes are diploid with cell walls composed mainly of β-1,3-D-glucans, β-1,6-D-glucans, and cellulose [5] with a small amount of chitin or chitosaccharides [6], [7], [8], [9]. Land plant parasitism has evolved independently in the Oomycota, possibly once in the Saprolegniales and at least twice in peronosporalean lineage [3]. To date, the genomes of six phytopathogenic species belonging to the peronosporalean lineage have been sequenced and annotated, including four species of the hemibiotroph *Phytophthora* (*Ph. ramorum*, *Ph. sojae*, *Ph. infestans*, and *Ph. capsici*) [2], [10], [11], the necrotroph *Pythium ultimum* var. *ultimum* [12] and the obligate biotroph *Hyaloperonospora arabidopsidis* [13].

*Pythium* is a polyphyletic group with over 250 species that has been organized into eleven phylogenetic clades based on multi-locus gene analysis [14], [15]. However, molecular studies indicate that the genus-level taxonomy of some of the clades is questionable [14], [16], [17]. For example, species belonging to clade K fit better in the description of the new genus *Phytopythium* than *Pythium* [15], [18], [19] and further taxonomic revisions within the genus are likely to be necessary [20]. *Pythium* spp. are biologically diverse and occupy different niches as saprophytes and as parasites of plants, fungi and animals [21], [22], including humans [23]. Phytopathogenic *Pythium* species are primarily necrotrophs that cause seed, root and fruit rots in a diverse range of species [21].

One barrier to plant colonization by microorganisms is the host cell wall which is composed predominantly of polysaccharides with lesser amounts of structural glycoproteins, phenolic esters, bound minerals, and enzymes [24]. The major polysaccharides present are cellulose, hemicellulose, and pectin. Hemicellulose includes xyloglucans, xylans, mannans, etc. [24]. The type of hemicellulose and the amount of pectin varies in the primary cell wall of different plants. Not surprisingly, degradation of the host cell wall is a key factor for pathogens or saprophytes invasion within plants [25], [26], [27], [28]. Moreover, some studies have associated the growth efficiency and aggressiveness of phytopathogens to their CAZyome, *i.e.*, the repertoire of predicted genes coding for carbohydrate-active enzymes (CAZymes) which are capable of degrading plant cell walls [29], [30], [31]. CAZymes are a general group of enzymes involved in the metabolism of carbohydrates and glycoconjugates and they include glycoside hydrolases (GH), glycosyl transferases (GT), polysaccharide lyases (PL), and carbohydrate esterases (CE) [32]. Carbohydrate-binding modules (CBM) contain carbohydrate-binding activity that is not part of the catalytic site and therefore CBM are indirectly associated with carbohydrate metabolism [32], and in this manuscript in order to simplify our results and discussions, they will be referred to as CAZymes. GH, CE and PL are deployed in the catabolism of carbohydrates based on the hydrolysis of glycosidic linkages of glycosides, de-O or de-N-acylation of substituted saccharides, and cleavage of uronic acid, respectively [32]. On the other hand, the transfer of sugar moieties accomplished by GT is associated with the synthesis of oligo and polysaccharides [32]. Therefore, with the exception of GT and CBM, CAZymes have been associated with plant cell wall degradation and consequently are considered pathogenicity factors [27], [30], [33].

In the companion paper published simultaneously in this issue, we described a comparative analysis of the genomes of seven *Pythium* spp., *Py. aphanidermatum* (*Pyap*), *Py. arrhenomanes* (*Pyar*), *Py. irregulare* (*Pyir*), *Py. iwayamai* (*Pyiw*), *Py. ultimum* var. *ultimum* (*Pyuu*), *Py. ultimum* var. *sporangiiferum* (*Pyus*), and *Py. vexans* (*Pyve*), with other plant pathogenic oomycetes and two diatoms in order to understand key genes and mechanisms involved in plant pathogenesis and necrotrophy in *Pythium* spp. (Adhikari, *et al.* companion paper, PLoS One, this issue). In this study, we detailed the genes involved in the degradation of plant cell walls and carbohydrate storage molecules. Genes encoding CAZymes are often overlooked in genome projects and faulty annotation may occur, especially due to the dual or broad substrate specificity nature of some enzymes [30], [34], [35], [36], [37] and because of the polyspecificity of some CAZyme families [32], [38]. We combined two different approaches for annotation, one uses sequence similarity (BLAST) [39] and PFAM domain-based searches (CAT) [40] and the other uses protein domain signatures examination (dbCAN) [41], both based on the Carbohydrate-Active EnZymes (CAZy) database [32], followed by manual verification of the genes. Here, we present the CAZyome of *Pythium* species and a broad comparative analysis with the CAZyomes of other plant pathogens belonging to the peronosporalean lineage. To corroborate our computational analyses, we measured growth of the seven *Pythium* species in minimal medium (MM) containing carbon sources typically present in plant cell wall and tissues. Our analysis revealed the interspecific diversity of the *Pythium*-CAZyomes and the comparison with the CAZyome of three *Phytophthora* species (*Ph. ramorum* (*Phra*), *Ph. sojae* (*Phso*), and *Ph. infestans* (*Phin*)) and *Hyaloperonospora arabidopsidis* (*Ha*) provided an indication on how CAZyme genes evolved in the peronosporalean lineage.

## Results and Discussion

### Phylogenetic relationships of species studied

**Figure 1:**
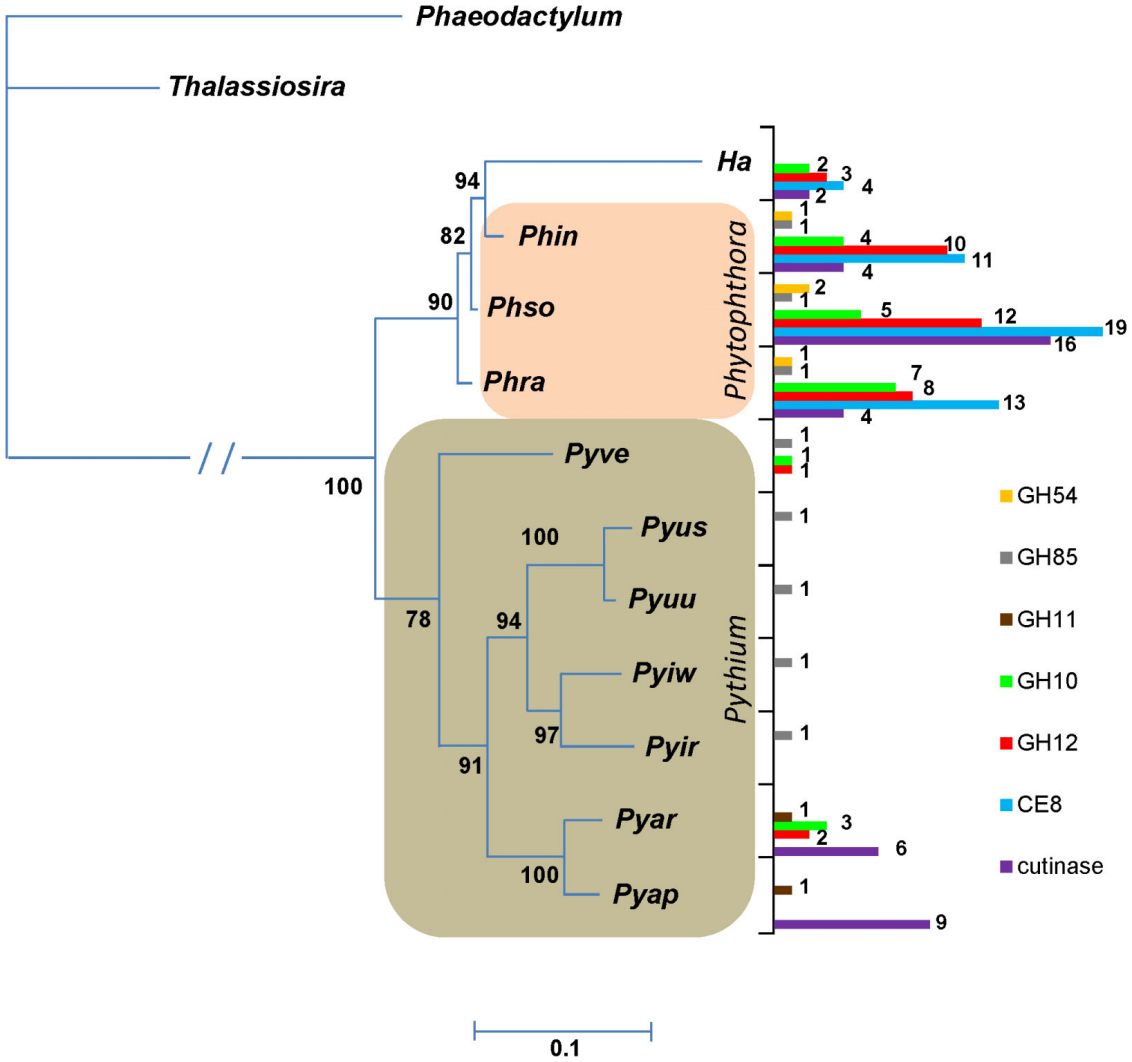
Phylogenetic tree of Stramenopiles species and distribution of CAZy genes in oomycete genomes. A Bayesian analysis was performed for 300,000 generations using a GTR/gamma distributed with invariant sites model of evolution of 28S rRNA gene. Bayesian probabilities are shown next to each branch. The distribution of seven gene families predicted from the genome of oomycete species and associated to carbohydrate degradation was compared to the phylogenetic relatedness thereof. Gene families: GH54 (orange), α-L-arabinofuranosidase; GH85 (gray), endo-β-N-acetylglucosaminidase; GH11 (brown) and GH10 (green), endoxylanases; GH12 (red), xyloglucan-β-1,4-D-endoglucanase; CE8 (blue), pectin methylesterase; and cutinase within CE5 (purple). Gene copy numbers are indicated next to the bars. Diatoms: *Phaeodactylum*, *Phaeodactylum tricornutum*; and *Thalassiosira*, *Thalassiosira pseudonana*. Oomycetes: *Ha*, *Hyaloperonospora arabidopsidis*; *Phin*, *Phytophthora infestans*; *Phso*, *Ph. sojae*; *Phra*, *Ph. ramorum*; *Pyve*, *Pythium vexans*; *Pyus*, *Py. ultimum* var. *sporangiiferum*; *Pyuu*, *Py. ultimum* var. *ultimum*; *Pyiw*, *Py. iwayamai*; *Pyir*, *Py. irregulare*; *Pyar*, *Py. arrhenomanes*; and *Pyap*, *Py. aphanidermatum*.

A Bayesian phylogenetic analysis based on the 28S rRNA gene of 11 Stramenopiles resulted in a diatom and an oomycete clade (Fig. 1). In the oomycetes, *Phytophthora* species were arranged in a monophyletic clade, having *Ha* as a sister group. *Pythium* species were distributed in two clades: one comprising *Pyve* which is closely related to *Phytophthora*, and the other containing two subclades, one of globose sporangial species (*Pyus*, *Pyuu*, *Pyiw* and *Pyir*), and one with filamentous sporangial species (*Pyar* and *Pyap*) (Fig. 1).

### Annotation of the CAZyome

Based on our analysis, the CAZyome of oomycetes corresponds to a range of 271 to 395 CAZymes in *Pythium* spp., 582 to 653 in *Phytophthora* spp. and 249 in *Ha* (Table 1). The enzymes were classified into superfamilies with GH the most abundant, followed by GT, CE, CBM and PL (Table 1, Table S1 and Fig. S1A). In order to verify the robustness of our annotation, we compared the CAZyome prediction of five species of *Pythium* (*Pyar*, *Pyir*, *Pyiw*, *Pyuu*, and *Pyve*) with their transcriptome sequences (RNA-seq) (Adhikari, *et al.* companion paper, PLoS One, this issue) [12]. The transcriptome of *Pyuu* was obtained from various conditions, including infection of plant (*Arabidopsis thaliana*) [12] and all the CAZyme-genes had expression support (Table S2). Expression analyses of the other species did not include plant infection (Adhikari, *et al.* companion paper, PLoS One, this issue) and their corresponding sequences covered 77.8 to 80.5% of *Pythium* CAZyomes (Table S2). Despite some discrepancies due to different strategies of gene annotation, the number of enzymes that we predicted for *Phytophthora* spp. and *Pyuu* using our method were similar to the values reported previously [12], [33]. The number of CAZymes was not correlated with genome size. The *Ha* genome (assembly of 81.6 Mb) [13] had the lowest number of CAZymes, whereas the *Pythium* genomes (33.9–44.7 Mb) had, on average, half the number of CAZymes as identified in the three *Phytophthora* genomes (65–240 Mb) [2], [10] (Table 1, Table S1 and Fig. S1A), suggesting gene expansion of the CAZymes in *Phytophthora* [33] compared to *Pythium* spp. (Adhikari, *et al.* companion paper, PLoS One, this issue) and *Ha* [13].

**Table 1:** Species of oomycetes and the corresponding carbohydrate-active enzymes (CAZymes) sorted according to the type of reaction catalyzed.

	*Pyap*	*Pyar*	*Pyir*	*Pyiw*	*Pyuu*	*Pyus*	*Pyve*	*Phra*	*Phso*	*Phin*	*Ha*
Assembly Size (Mb)*	35.9	44.7	43.0	43.2	42.8	37.6	33.9	65	95	240	81.6
CBM	38	34	35	45	46	24	36	55	53	45	21
GH	115	157	134	124	161	117	156	266	293	261	100
GT	105	113	102	105	96	85	104	125	138	142	81
PL	21	5	15	7	29	15	21	44	51	57	12
Cutinase	9	6	0	0	0	0	0	4	16	4	2
PME	0	0	0	0	0	0	0	13	19	11	4
other CE	61	73	59	48	63	30	56	75	83	66	29
total	349	388	345	329	395	271	373	582	653	586	249

**Oomycete species**: *Pyap* = *Pythium aphanidermatum*; *Pyar* = *Py. arrhenomanes*; *Pyir* = *Py. irregulare*; *Pyiw* = *Py. iwayamai*; *Pyuu* = *Py. ultimum* var. *ultimum*; *Pyus* = *Py. ultimum* var. *sporangiiferum*; *Pyve* = *Py. vexans*; *Phra* = *Phytophthora ramorum*; *Phso* = *Ph. sojae*; *Phin* = *Ph. infestans*; and *Ha* = *Hyaloperonospora arabidopsidis*.

**CAZymes categories**: CBM = carbohydrate-binding modules; GH = glycoside hydrolases; GT = glycosyl transferases; PL = polysaccharide lyases; PME = pectin methyl esterase; other CE = carbohydrate esterases excluding cutinases and PME; total = total number of CAZymes.

* Assembly genome sizes were according to data published by: Lévesque *et al.* for *Pyuu* [12], Adhikar *et al.* for the other species of *Pythium* (Adhikari, *et al.* companion paper, PLoS One, this issue), Haas *et al.* for *Phin* [10], Tyler *et al.* for *Phra* and *Phso* [2], and Baxter *et al.* for *Ha* [13].

CAZyme superfamilies were subsequently classified into families based on the structural features of the enzymes, according to the CAZy classification scheme [32]. A range of 82–87, 85–88 and 72 CAZyme families were annotated in the *Pythium*, *Phytophthora*, and *Ha* genomes, respectively (Table S1 and Fig. S1B), with most of the families shared among all oomycetes, with some exceptions (Table S1). The more abundant CAZyme families and families with more impact on plant cell wall and storage degradation are detailed below. Growth of *Pythium* in media containing selected carbon sources supported most of our predictions.

### Degradation of plant polysaccharides

#### Cellulose metabolism

Cellulose consists of a linear chain of several β-(1-4) linked D-glucose units and is one of the main constituents of oomycete and plant cell walls [24]. In *Phytophthora*, cellulose is also involved in appressorium formation and consequently in pathogenicity [42]. All oomycete genomes analyzed to date, including the seven *Pythium* species examined in this study, have one to three copies of cellulose synthase genes within the GT2 family which is associated with oomycete cell wall synthesis. However, the cellulolytic efficiency of *Pythium* spp., *i.e.* the ability to degrade cellulose from the plant, is controversial and variable among isolates of the same species [29], [43], [44], [45], [46], [47], [48], [49], [50], [51]. For this reason, *Pythium* spp. were once referred to as “sugar fungi” as they were believed to be unable to degrade complex structural polymers [48], [52].

**Figure 2:**
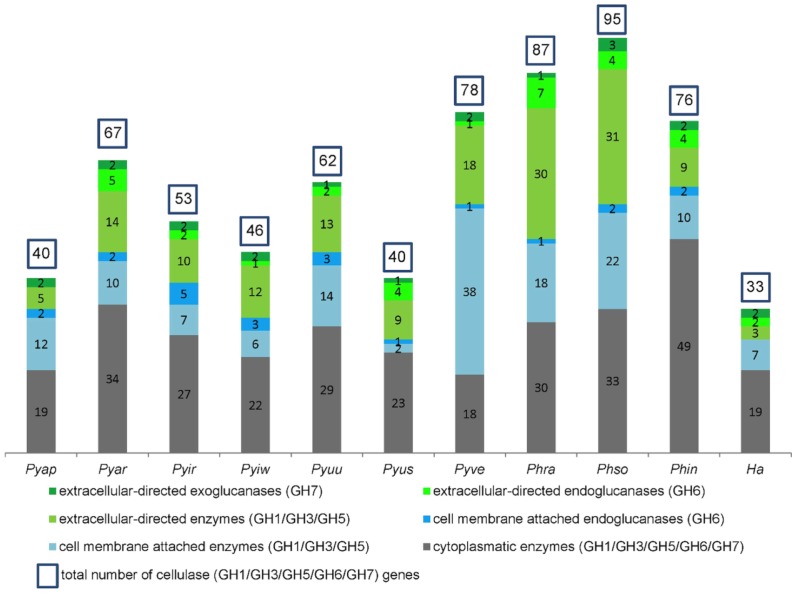
Glycoside hydrolase (GH) families associated with cellulose metabolism. Families GH1, GH3 and GH5 are cellulase candidates, *i.e.*, they may or may not be related to cellulose metabolism. Genes belonging to GH6 and GH7 encode enzymes that are strictly related to cellulose metabolism, either to the oomycete cell wall (membrane attached) or to the plant cellulose catabolism (extracellular directed). Species abbreviations are as defined in Figure 1.

**Figure 3:**
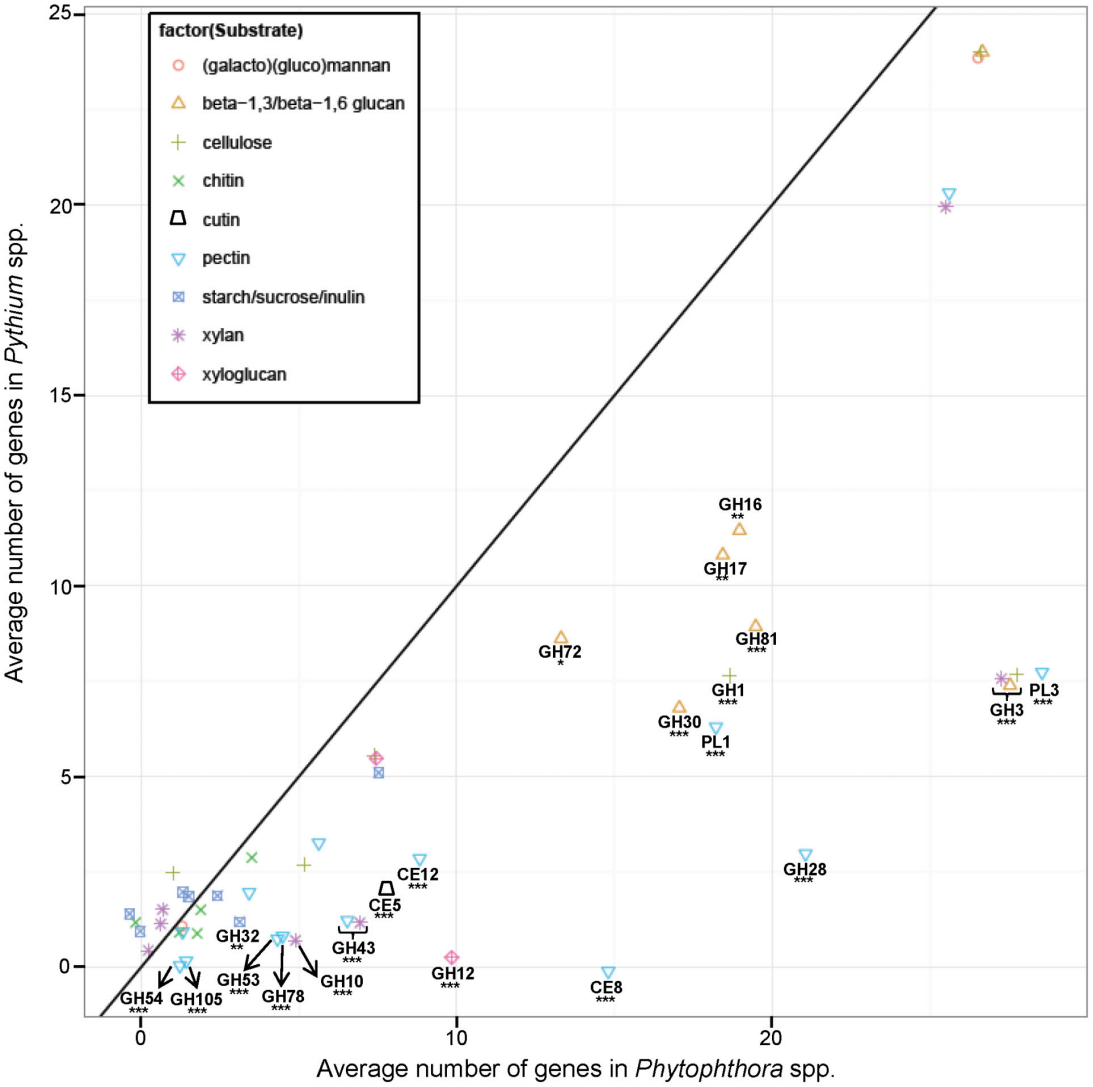
Average copy number of some CAZyme-gene families in *Pythium* and *Phytophthora* sorted by substrate. Black line corresponds to the equal number of copies in *Pythium* and *Phytophthora*. Based on the GLM (loglinear/Poisson) test all gene families whose number of copies is significantly more abundant in *Phytophthora* than in *Pythium* are indicated: ***, ** and * represent p<0.001, p<0.01 and p<0.05, respectively.

Cellulases are classified into endo-β-1,4-D-glucanases, cellobiohydrolases (exocellulases), and β-1,4-glucosidases [38], [53], [54], [55], [56], [57]. Based on the CAZy database [32], eight GH families contain potential cellulases which have been annotated in eukaryotic genomes. Based on our method, three of these, GH9, GH45 and GH48, were not detected in the 11 surveyed oomycete genomes, but GH5, GH6 and GH7 which encode endocellulases and cellobiohydrolases, as well as GH1 and GH3 which encodes β-glucosidases (Table S1 and Fig. 2), were present. GH1, GH3 and GH5 were highly represented in all oomycete genomes (Table S1 and Fig. S1B), but since these families have members that are capable of degrading distinct substrates [30], [32], [34], [35], [36], [38], subsequent enzyme characterization would be necessary to determine if they indeed act on cellulose, whether GH6 and GH7 genes only encode cellulase. We determined if the predicted cellulases were directed to the oomycete cell wall metabolism (membrane-attached cellulases) or exported and likely associated with plant cell wall degradation (extracellular-directed cellulases), based on the analysis of secretion signals, transmembrane domains, and glycosylphosphatidylinositol (GPI) anchors (Fig. 2). *Phytophthora* spp. had, on average, more genes potentially related to cellulose metabolism than *Pythium*, while *Ha* had the lowest number (Fig. 2 and Table S1). Some of the GH families were significantly more represented in *Phytophthora* genomes, which may be a result of a selective expansion of those genes (Fig. 3). Based on their predicted cellular location, the majority of the genes are probably associated to the oomycete cell wall metabolism. In contrast, the number of extracellular-directed exo- and endoglucanases (GH6 and GH7) genes (2–8 copies) that are deployed in plant cellulose degradation is low in all oomycetes (Fig. 2). Some monooxygenases known to enhance the breakdown of cellulose [58], [59] were also detected in most of the genomes (Table S1), and despite not encoding glycosidases, they are traditionally classified as GH61 by the CAZy database [32].

The seven species of *Pythium* were incubated on MM plates containing cellulose and cellobiose (Table 2). The *Pyar* genome encodes the highest number of extracellular-directed GH6 and GH7 cellulases (7) of all the *Pythium* spp. (Figure 2) and its growth in cellobiose was high, yet limited in MM containing cellulose as the sole carbon source (Table 2). In fact, limited growth in cellulose was observed in all *Pythium* species. These data suggest that most of the genes encoding cellulase are associated with oomycete cell wall metabolism (Fig. 2) and that there is limited capability of *Pythium* to degrade cellulose (Table 2), sufficient to facilitate hyphal penetration into plant cell walls yet not enough to provide complete digestion of plant cellulose as a carbon source [12], [49]. These results are consistent with the observation that *Pythium* spp. preferentially colonize young root tissues such as root hairs or root tips which lack complex polymers [60], [61], [62].

**Table 2:** Proportional growth of *Pythium* species on a minimal medium (MM) containing various carbon sources to its growth on V8 juice agar.

Clade*	(A)	(B)	(F)	(G)	(I)	(I)	(K)
Species	*Pyap*	*Pyar*	*Pyir*	*Pyiw*	*Pyuu*	*Pyus*	*Pyve*
							
**Control**							
V8 juice agar	100**	100	100	100	100	100	100
MM	4.0±1.0	6.0±2.1	3.3±0.3	6.0±1.3	1.3±0.3	7.3±1.3	1.3±0.3
**Monosaccharides**							
D-glucose	20.3±1.3	100	10.0±1.0	29.7±1.3	100	100	12.7±1.7
D-fructose	20.3±1.3	100	8.7±0.3	15.7±0.7	100	100	3.0±1.5
D-galactose	5.7±1.5	25.7±1.5	16.7±1.5	10.3±2.4	1.0±0.6	16.3±1.7	2.3±0.3
D-mannose	15.7±5.5	2.3±0.3	25.0±2.0	2.3±1.3	24.3±3.0	11.7±0.8	13.7±1.3
D-xylose	2.3±0.3	9.0±1.5	7.3±2.6	17.6±1.3	5.6±1.8	24.3±1.3	6.7±2.4
L-arabinose	14.7±1.7	16.3±3.4	19.0±4.0	18.7±0.8	1.3±0.3	8.3±0.8	4.7±0.7
L-rhamnose	14.7±1.8	10.0±1.5	19.0±4.0	19.3±0.3	1.3±0.3	5.3±0.3	3.3±0.7
**Disaccharides**							
cellobiose	15.3±0.8	100	11.7±0.3	19.3±1.2	68.7±3.7	81.7±4.4	17.0±1.1
sucrose	21.0±2.0	100	11.3±0.6	36.7±2.7	100	100	5.3±0.7
**Uronic acid**							
D-galacturonic acid	2.7±1.5	3.7±1.2	9.3±1.2	10.3±0.3	4.7±1.7	5.7±0.7	1,3±0.7
**Polysaccharides**							
beechwood xylan	6.3±1.8	28.7±2.9	25.0±0.6	11.6±0.3	24.7±2.9	17.7±1.5	0
guar gum	57.3±5.9	1.3±0.7	75.7±3.0	0	100	100	0
starch	69.0±2.3	100	39±2.8	72.3±2.3	100	100	100
pectin	8.7±1.2	100	24.7±0.9	0.7±0.3	100	18.0±1.1	11.3±1.9
cellulose	30.7±2.0	11.7±1.7	19.7±0.3	25.7±2.7	20.7±1.2	0.3±0.3	0

* Clades denominations are based on Lévesque & de Cock [1414].

Species: *Pyap* = *Pythium aphanidermatum*, *Pyar* = *Py. arrhenomanes*, *Pyir* = *Py. irregulare*, *Pyiw* = *Py. iwayamai*, *Pyuu* = *Py. ultimum* var. *ultimum*, *Pyus* = *Py. ultimum* var. *sporangiiferum*, and *Pyve* = *Py. vexans*.

** Values represent proportional diameter growth means of each isolate on carbon sources relative to its growth on V8 juice agar (± standard error).

#### Xyloglucan degradation

**Figure 4:**
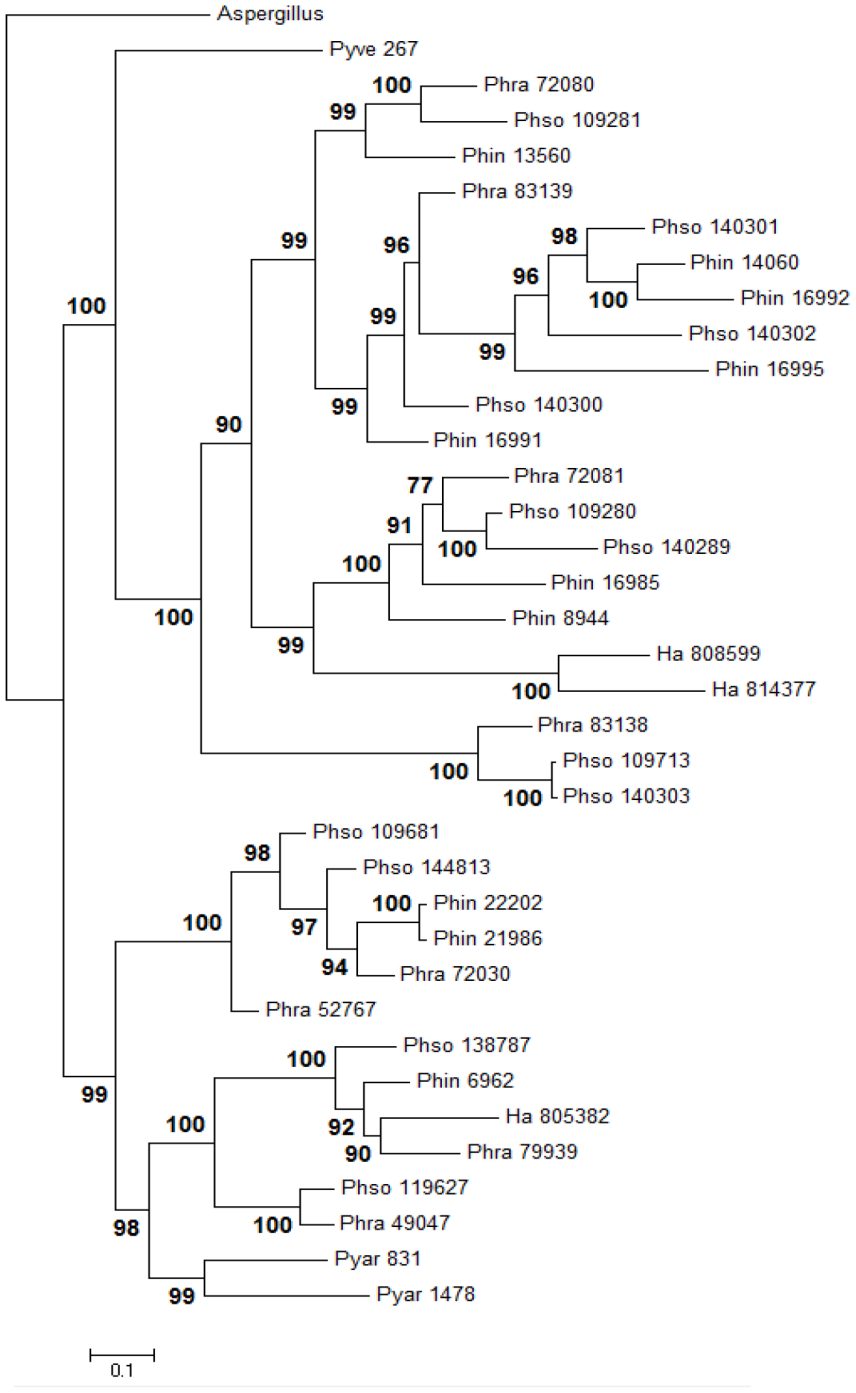
Phylogenetic relationship among predicted xyloglucan-β-1,4-D-endoglucanases (GH12) of oomycetes. Bayesian analysis was performed for 300,000 generations using Blosum model of evolution. Bayesian probabilities are shown next to each branch. An endoglucanase of *Aspergillus clavatus* (XP_001269687) was used as outgroup. Leaves indicate the locus number of predicted proteins in the genomes of each species (as defined in Figure 1).

Xyloglucan is the most common hemicellulose polysaccharide in the primary cell wall of non-graminaceous plants [24]. It is associated with cellulose microfibrils and therefore adds structural integrity to the cell wall [63]. Due to the nature of its backbone, endoglucanases and β-glucosidases involved in cellulose degradation are also able to degrade xyloglucan [63]. However, a set of enzymes is exclusively associated in xyloglucan degradation. For example, GH74 and GH29/GH95, which are associated with xyloglucan-β-1,4-D-endoglucanase and α-L-fucosidase activity, respectively, were not detected by us in the surveyed oomycete genomes. The absence of GH95 in *Phytophthora* genomes is in disagreement with Ospina-Giraldo and collaborators [33], probably due to different methods of gene prediction and annotation chosen by the two groups. In contrast, we detected xyloglucan-β-1,4-D-endoglucanase genes in family GH12 in *Ha*, *Phytophthora* spp., and *Pyve* and *Pyar* (Fig. 1 and Table S1). The occurrence of these genes in genus-specific clades (with the exception of *Ha* 805382) (Fig. 4) is consistent with previous reports [13], [64]. GH31 genes were annotated in *Pythium* spp. (4–6), *Phytophthora* spp. (5–9) and *Ha* (3) (Table S1), and based on sequence similarity with Swiss-Prot database entries, encode both α-D-xylosidases and α-glucosidase [65], [66]. Therefore, further enzyme characterization would be necessary to confirm their substrate specificity.

The xyloglucan degradation capacity of *Pythium* spp. appears to be restricted or totally absent [12], [62]. Some of the GH12 genes present in the surveyed *Phytophthora* spp. genomes appear to have been lost in the other oomycetes (Fig. 1), consistent with observations reported in Baxter *et al.* [13]. Furthermore, the expanded number of GH12 genes in *Phytophthora*, probably due to duplication events as first inferred by Costanzo *et al.* [64], is more evident when compared to the number of copies in *Pythium* (Fig. 3 and 4). The difference in number may confer a greater ability of *Phytophthora* to proliferate in plant tissue relative to *Pythium* [64].

#### Xylan degradation

Xylan is abundant in the primary cell wall of grasses and in the secondary cell wall of woody plants, but is a minor component of the primary cell walls of dicotyledons and non-graminaceous monocotyledons [24]. Xylan degradation depends primarily on two enzymes, endo-β-1,4-xylanase and β-1,4-D-xylosidase [63]. Xylanase activity was reported at low levels only in *Py. sulcatum* [29], but neither enzyme activity [29] nor genes exclusively related to xylanase degradation were reported in *Py. ultimum* [12]. In our studies, two families of endoxylanases were detected: GH10 in *Ha*, *Phytophthora* spp., *Pyve*, and *Pyar*; and a single copy of GH11 in *Pyar* and *Pyap* (Fig. 1 and Table S1). *Pyve* is closely related to *Phytophthora* species [18] and *Pyar* and *Pyap* are part of a separate clade of *Pythium* species (Fig. 1). The presence of endoxylanases in *Pyar* and *Pyap* corroborates the xylanase activity detected in *Py. sulcatum* [29], a species that is also part of the filamentous sporangial clade [14], [15].

**Figure 5:**
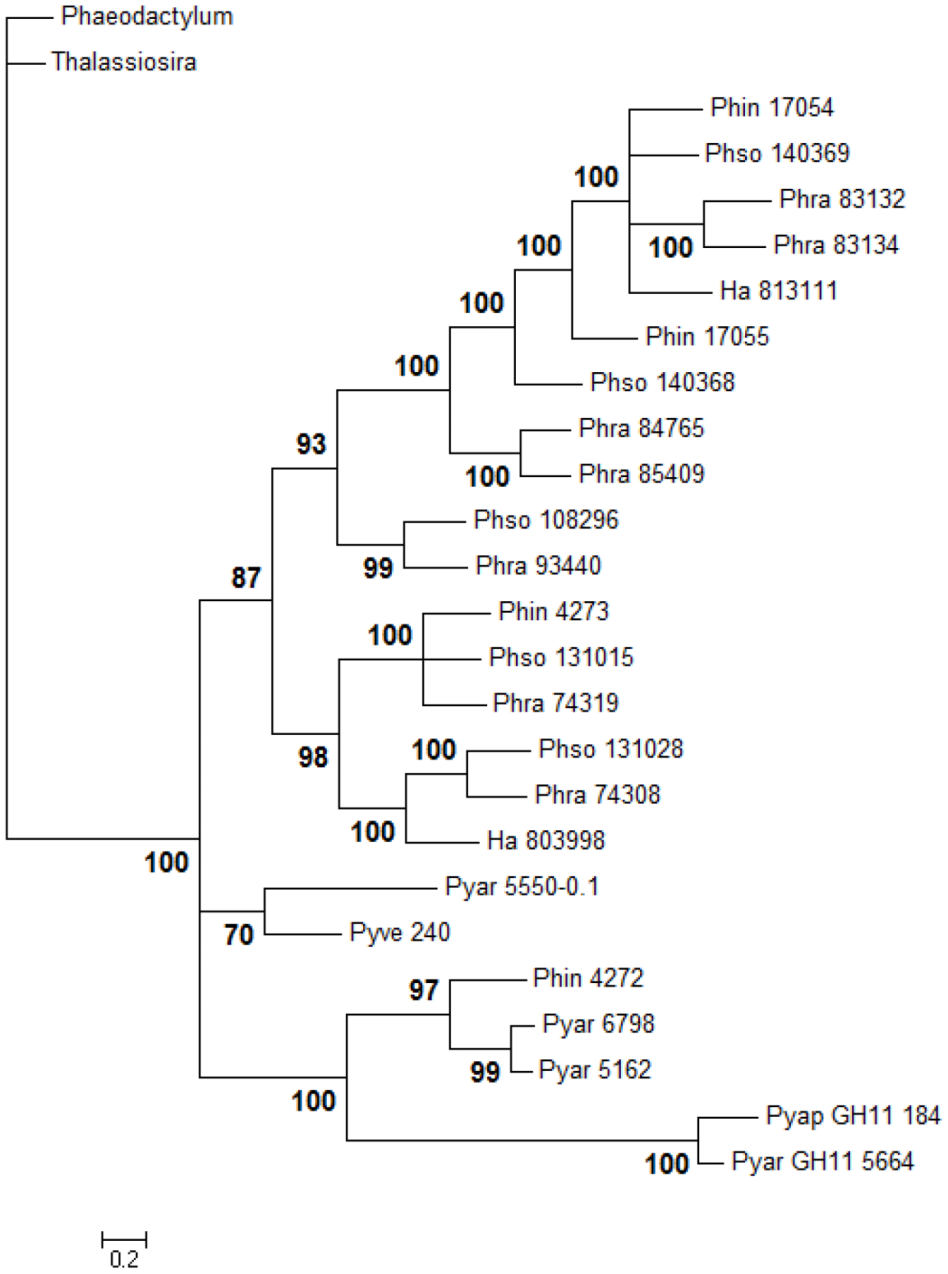
Phylogenetic relationship among predicted endoxylanases (GH10 and GH11) of straminipilous species. Bayesian analysis was performed for 300,000 generations using Blosum model of evolution. An endoxylanase of *Phaeodactylum tricornutum* (XP_002178502) and *Thalassiosira pseudonana* (XP_002290930) were used as outgroups. Bayesian probabilities are shown next to each branch. Leaves indicate the predicted proteins: species abbreviations (as defined in Figure 1), and locus number within the corresponding genome. All entries correspond to GH10 endoxylanases, unless represented as GH11.

Phylogenetic analysis indicated that endoxylanases from GH10 family clearly differ from GH11 (Fig. 5). GH10 was probably present in a common ancestor of Stramenopiles and underwent expansion in *Phytophthora* [33] (Fig. 3), but was lost in most of *Pythium* species and *Ha*. In contrast, GH11 was most likely lost in all Stramenopiles but was maintained in *Pyar* and *Pyap*, which may be a characteristic of all filamentous sporangial species of *Pythium*. Interestingly the GH11 gene in *Pyar* and *Pyap* is present in a 1.5 kb-contig and 48 kb-scaffold respectively that are not shared with other Stramenopile genomes.

Overall, the number of endoxylanase genes present in oomycete genomes was low compared to the predicted number (14) in *Podospora anserina* [38], an ascomycete that degrades cellulose, xylan, and lignin. Genes of GH3 family were abundant (Table S1) with some annotated as β-1,4-D-xylosidases. However, it is difficult to infer based only on *in silico* analysis if they are involved in xylan or in other substrate metabolism [29], [30], [34], [35], [36]. Some genes coding for xylan side chain removal or modification belonging to carbohydrate esterase families (CE1, CE2 and CE3) were automatically detected by hidden Markov models [41], while other genes were absent (*e.g.* GH62, GH67, and GH115) (Table S1).

CAZyome analysis indicating that *Pythium* species may be inefficient xylan degraders is consistent with *in vitro* growth studies in which most of the species had limited growth in a medium containing xylan (Table 2).

#### (Galacto)(gluco)mannan degradation

Mannose-containing polysaccharides are composed of a 1,4-β-linked D-mannan backbone that may be interspersed with 1,4-β-linked D-glucose and substitutions of 1,6-α-linked D-galactosyl [63]. Galactomannans are most commonly found in the seeds of Leguminosae, and glucomannans are abundant in the secondary cell wall of woody plants whereas galactoglucomannans are generally found in both primary and secondary plant cell walls and are the principal hemicelluloses in the wood of gymnosperms [24], [63], [67]. Oomycete genomes encode few genes related to (galacto)(gluco)mannan degradation (Table S1). Based on the CAZy database, the GH2, GH5 and GH26 families comprise β-mannosidase and β-mannase among other enzymes [32]. GH26 genes were not detected in any oomycete genome and eight GH5 genes (one copy in *Pyve* and *Ha*, and two copies in *Phra*, *Phso*, and *Phin*) were assigned as β-mannosidases, after compared to the Swiss-Prot database. In contrast, with the exception of *Ha*, all genomes contained one copy of a GH2 gene coding for β-mannosidase (PTHR10066:SF12) (Table S1). No α–galactosidase gene candidates (GH27 and GH36) were detected in the oomycete genomes. Based on studies of true Fungi, the low number of genes detected in *Pythium* may be sufficient for mannan degradation [38], [68], [69].

None of the *Pythium* species grew well in a medium containing D-mannose (Table 2), but *Pyir*, *Pyuu* and *Pyus* did exhibit robust growth on guar gum (galactomannan). Accordingly, these species are consistently associated with seed rot and damping-off in leguminous plants that are rich in galactomannan [70], [71], [72], [73], [74]. However, we did not detect variation in the number of predicted genes in *Pyir*, *Pyuu* and *Pyus* (Table S1), hypothetically deployed in galactomannan degradation, relative to the other *Pythium* species, that would explain their growth on these carbohydrate sources.

#### Pectin degradation

**Figure 6:**
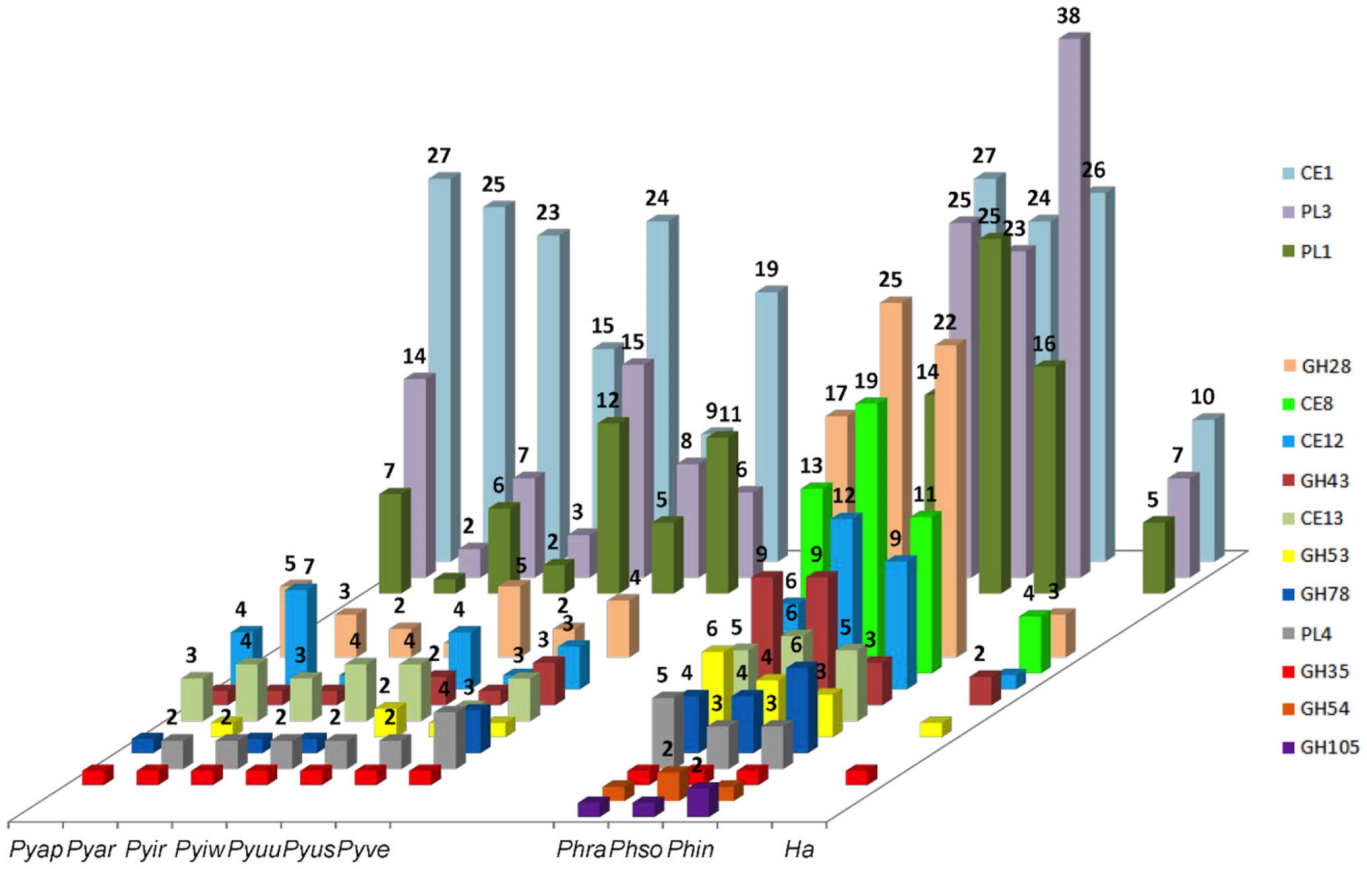
Pectin degrading enzymes in oomycetes. Bars correspond to one gene copy, unless indicated. Carbohydrate esterase (CE), pectin/pectate lyase (PL) and glycoside hydrolase (GH) gene families: CE1 = feruloyl esterase and others; PL3 = pectate lyase; PL1 = pectin/pectate lyase; GH28 = polygalacturonase; CE8 = pectin methyl esterase; CE12 = pectin acetylesterase; GH43 = endo-1,5-α-L-arabinosidase and β-xylosidase; CE13 = pectin acetylesterase; GH53 = endo-β-1,4-galactanase; GH78 = α-L-rhamnosidase; PL4 = rhamnogalacturonan lyase; GH35 = β-galactosidase; GH54 = arabinofurosidase and β-xylosidase; GH105 = unsaturated rhamnogalacturonyl hydrolase. Species abbreviations are as defined in Figure 1.

Pectins are complex heteropolysaccharides that are important components of the plant cell wall and middle lamella [24]. The simplest type of pectin is composed of an α-1,4-linked D-galacturonic acid backbone that can be acetylated or methylated. In other types of pectin, the backbone presents substitutions of D-xylose or is interrupted by residues of L-rhamnose, in which arabinan and galactan chains can be attached [63], [75]. Polygalacturonases break down the 1,4-glycosidic linkage of pectin and pectates and are considered to be the primary cause of tissue maceration in soft rot diseases [46]. In *Ph. infestans*, polygalacturonases were expressed during both pre-infection and infection stages [76] and in *Ph. parasitica*, expression of this enzyme was clearly linked to pathogenicity [77]. The pectin backbone is essentially disrupted by GH28 enzymes, comprised of exo- and endo- polygalacturonases and rhamnogalacturonases, and pectin lyase families 1, 3 and 4, which encode pectin/pectate lyases, pectate lyases and rhamnogalacturonan lyases, respectively. All of these families were present in the *Phytophthora* genomes [2], [10], [33] and most were also present in much lower numbers in *Pythium* spp. and *Ha* (Table 1, Table S1 and Fig. 6). In contrast, *Phytophthora* genomes encode most of the enzymes targeting different residues and side chains, whereas the other genera lack some of these genes (Table S1 and Fig. 6). Genes encoding unsaturated rhamnogalacturonyl hydrolase (GH105) and d-4,5-unsaturated β-glucuronyl hydrolase (GH88) involved in saccharification of the products of pectin and pectate lyases were not detected in any of the *Pythium* genomes or *Ha*, whereas GH105 was present in all *Phytophthora* species (Fig. 6). *Pythium* spp., unlike *Phytophthora* and *Ha*, lack pectin-methylesterase activity [29], [43], [45], [46] and accordingly genes of CE8 family were detected in *Ha* [13] and *Phytophthora* [33], but not in *Pythium* (Table 1, Fig. 1 and Fig. 6). Baxter *et al.* [13] proposed that some copies of pectin-methylesterase genes were lost in *Ha* and we believe that a complete gene loss occurred in a common ancestor of *Pythium*. In contrast, these genes probably underwent gene expansion in *Phytophthora* spp. Ten CAZy families involved in pectin degradation were significantly more abundant in *Phytophthora* spp. than in *Pythium* spp. (Fig. 3) which may be an indication of a biased duplication of pectin-related genes in the genomes of *Phytophthora* species [2], [10], [33] (Adhikari, *et al.* companion paper, PLoS One, this issue).

Pectin degradation has been reported in many *Pythium* species [29], [43], [45], [46], [49], [51] and our genome analysis and carbon utilization studies support this conclusion (Table 2). One exception was *Pyiw*, a weakly pathogenic species that is capable of growing and parasitizing plants at low temperatures. It had the lowest number of predicted pectin degrading enzymes (Table S1 and Fig. 6) and accordingly, its growth in medium containing pectin was minimal. Growth of *Pythium* in media containing monosaccharides commonly present in the pectin side chains or backbone substitutions (D-galactose, L-arabinose, D-xylose and L-rhamnose) was limited (Table 2), which support our hypothesis that pectin degradation is important for *Pythium* to gain access to plant cells [12], [49], [62], but not for the complete saccharification of this complex sugar.

#### Cutin degradation

**Figure 7:**
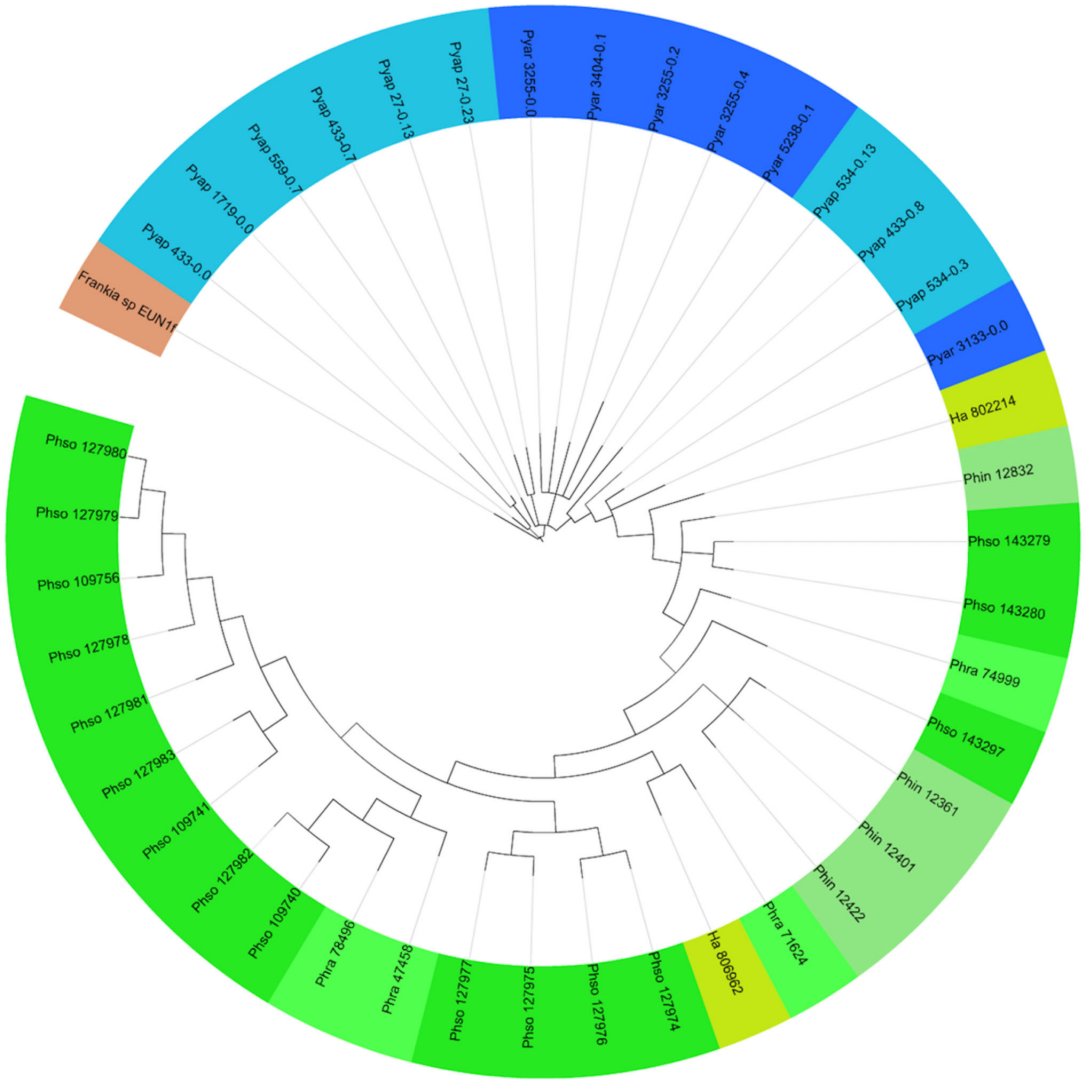
Phylogenetic relationship among predicted cutinases of oomycete species. The cutinase encoding genes identified from *Hyaloperonospora arabidopsidis*, *Phytophthora* spp. and *Pythium* spp. genomes were used for the phylogenetic analyses. A cutinase sequence from *Frankia* sp. EUN1f (ZP_06415970) was used as outgroup. The phylogeny was inferred using using Blosum model of evolution (300,000 generations) and displayed using the Interactive Tree of Life (iTOL) web server (http://itol.embl.de/). The same color indicates cutinase from the same genus, different shades indicate different species. Species abbreviations are as defined in Figure 1.

Cutin is a polymer of hydroxyl fatty acids that is especially present on cells that cover the aerial surfaces of higher plants [78]. Cutin degradation facilitates the penetration of suberized roots, leaves and stem tissues [12]. Cutinase activity was previously reported in *Phytophthora* species [33], [79], [80], [81], *Ha* [13], *Pyap*, and *Pyar* [82], but little or no activity was detected in *Pyuu* [29], [82]. In *Ph. infestans*, cutinase genes were up-regulated during host infection, but no conclusive association between pathogenicity and cutinase activity was demonstrated [83]. We identified cutinase-encoding genes (IPR000675) within the CE5 family in *Ha*, *Phytophthora* spp., and in *Pyar* and *Pyap* (Table 1, Table S1 and Fig. 1) (Adhikari, *et al.* companion paper, PLoS One, this issue), consistent with cutinase activity reports. The cutinases present in *Phytophthora* spp. and *Ha* were clustered in a monophyletic group, contrary to the cutinases from *Pythium* spp. genomes (Fig. 7), reflecting the polyphyletic nature of the latter group [14], [15]. Our phylogenetic analysis (Fig. 7) confirms the gene expansion described in *Phytophthora* [83] and also unveils the same pattern in *Pyap* and *Pyar*. The globose sporangial *Pythium* species likely lost cutinase genes, while they were maintained in the filamentous-sporangial species, similar to what was observed with the GH11-endoxylanase and xyloglucan-specific-endoglucanase genes (Fig. 1).

#### Starch, sucrose and inulin degradation

Starch is an α-1,4-linked D-glucose polymer which is the most important reserve carbohydrate in the majority of higher plants [84] and inulin, a polymer composed mainly of fructose with a terminal glucose, is alternatively used by some plants in roots and rhizomes [85]. Sucrose, a non-reducing disaccharide of an α-D-glucose and β-D-fructose, is the major form for carbon translocated from photosynthetic cells [84].

The oomycetes have a set of enzymes dedicated to starch, inulin and sucrose degradation. Genes encoding α-glucosidase (GH31) are involved in both starch and sucrose metabolism and are present in all oomycete genomes (Table S1). Genes encoding α-amilase (GH13), α-glucoamylase (GH15) and N-terminal starch-binding modules (CBM20, 21, and 25) are associated with starch metabolism and are present in most of the genomes (Table S1). β-fructosidase (GH32), which may be associated with sucrose and inulin catabolism, was represented by one copy in all *Pythium* genomes and by three copies in *Phytophthora* spp. and *Ha*. The number of genes involved in starch, sucrose and inulin degradation (excluding CBM) in *Ha* (7), *Pythium* (7–11), and *Phytophthora* (10–13) spp. (Table S1), is generally lower than in true Fungi (11–37) [38]. While *Phytophthora* species are more equipped with enzymes acting in the degradation of complex carbohydrates than *Pythium*, the majority of the genes involved in the degradation of simple sugars are uniformly distributed in all oomycetes genomes (Fig. 3). Most of *Pythium* species had a robust growth in starch and sucrose (Table 2), which make these plant saccharides the primary carbon source for growth.

### Metabolism of other polysaccharides

#### Chitin and chitosan metabolism

The number of genes related to chitin or chitosaccharides metabolism in oomycete genomes is limited, in agreement with previous reports [9], [12], [49], [62]. This is consistent with the rarity of chitin in oomycete cell walls [86] and its absence in plant cell walls. Chitinase-encoding genes within the families GH18 or GH19, chitin deacetylases (CE4), and N-acetylglucosamine 6-phosphate deacetylases (CE9) were present in low numbers in the oomycete CAZyomes, but CE9 was only detected in *Pythium* spp. (Table S1). One copy of GH85 which encodes endo-β-N-acetylglucosaminidase was annotated in all genomes, except *Pyap*, *Pyar* and *Ha* (Fig. 1 and Table S1). Genes belonging to families GH20 and GH48, encoding β-1,6-N-acetylglucosaminidase and chitinase, respectively, were absent. The number of genes encoding chitin synthase within GT2 ranged from one copy in *Ha* to 1–2 in the *Pythium* and *Phytophthora* species. No CBM18 (chitin-binding modules) was identified (Table S1). No genes exclusively associated with chitosan metabolism (GH46, GH75 and GH80) were detected. It would be interesting to compare the number of chitin-related genes in the genomes of the mycoparasitic *Py. oligandrum* and the flesh eating *Py. insidiosum*.

#### β-1,3 and β-1,6 glucan metabolism

Callose, a polysaccharide composed of β-1,3-linked glucans, is only synthesized in plants during cytokinesis, pollen development, and in response to stress [87]. However, the bulk of oomycete hyphal walls are composed of β-1,3- and β-1,6-glucans [88]. Mycolaminaran, the polysaccharide form of storage in oomycetes, is also composed of β-1,3-glucan chains [89]. All genomes contain multiple copies of endo-1,3-β-glucanase and glucan 1,3-β-glucosidase within the GH16 and GH17 families (Table S1 and Fig. 3). β-1,3-glucanosyltransglycosylases (GH72) and (endo-)β-1,3-glucanases (GH81), which are associated with the modification and cross-linkage of linear β-1,3-glucans, are also abundant (Table S1 and Fig. 3). Similarly, oomycetes genomes are rich in genes encoding for 1,3-β-glucan synthase (GT48) (7–13 copies) (Table S1), consistent with the hypothesis that the majority of this class of genes is associated with the oomycete cell wall metabolism or carbohydrate storage, rather than degradation of plant β-1,3-glucan. GH30 genes can be associated with the metabolism of β-1,6-glucanases, glucosylceramidase, β-xylosidase and others [90] and this family was abundant in all oomycete genomes (Table S1), particularly in *Phytophthora* spp. (Fig. 3). A parallel analysis showed that some families related to β-1,3 and β-1,6 glucan metabolism were either enriched in the *Pythium* or *Pythium*-*Phytophthora* specific gene set or depleted in the genomes of photosynthetic Stramenopiles, when the gene arsenal of oomycetes and diatoms were compared (Adhikari, *et al.* companion paper, PLoS One, this issue).

## Conclusion

*Pythium* spp. are called “sugar fungi” because they degrade simple carbohydrate polymers [48], [52] and apparently only rely on them for growth. The number of genes related to starch and sucrose catabolism is considerably uniform among *Pythium* and other oomycete genomes. In contrast, *Pythium* species cannot completely metabolize the complex carbohydrate constituents of plant cell wall as an energy source. Instead, these pathogens produce pectinases, and some species cellulases, to partially macerate cell walls in order to gain access to simple sugars within plant cells. Few *Pythium* genes are involved in the metabolism of xyloglucan, xylan, mannose, and cutin. In fact, the variable distribution of these genes in different species of *Pythium* coincides with the polyphyletic nature of the genus [14], [15], [18]. Genes related to the oomycete cell wall metabolism are predominant in all CAZyomes, but differential expansion of some classes of genes was observed in the genomes of oomycetes. Compared to other oomycetes, the *Pythium* CAZyome did not undergo expansion as in *Phytophthora* [33] and some CAZy-encoding genes were lost in all or a subset of *Pythium* species [91], but not to the same magnitude as in *Ha* [13]. The CAZyome repertoire related to plant sugars degradation probably has an important influence on *Pythium* lifestyle as a primary plant pathogen. When infecting plants, *Pythium* rapidly degrades simple carbohydrates within plant cells and then allocates its metabolic resources to reproductive and survival structures (oospores) to facilitate reproduction and dissemination, rather than competing for microbial degradation of cellulose, hemicelluloses and pectin.

## Materials and Methods

### Isolates, DNA sequencing and gene prediction

The isolates used in this study were: *Pyap* (CBS 132490 = DAOM BR444), *Pyar* (CBS 324.62 = ATCC 12531), *Pyir* (CBS 250.28 = DAOM BR486), *Pyiw* (CBS 132417 = DAOM BR242034), *Pyuu* (CBS 805.95 = DAOM BR144 =  ATCC 200006), *Pyus* (CBS 219.65 = DAOM BR650), and *Pyve* (CBS 119.80 = DAOM BR484). DNA sequencing and gene prediction are described in Adhikari et al. (companion paper, PLoS One, this issue).

### Sole carbon source growth experiment

Each isolate was grown in V8 juice agar medium [92] for 2–3 days. A 4-mm-diameter plug containing vigorously growing mycelium was excised and placed in the center of a Petri dish containing minimal medium [93] in 1.5% agarose or amended with a sole carbon source. The carbon sources were beechwood xylan, guar gum, soluble starch, pectin and cellulose at 1% (w/v), and D-glucose, D-fructose, D-galactose, D-mannose, D-xylose, L-arabinose, L-rhamnose, D-galacturonic acid, cellobiose and sucrose at 25 mM (Table S3). The pH was adjusted to 6.0 and the media were autoclaved at 121°C for 25 minutes. Isolates were grown in three replicates for each of the carbon source and incubated at 25°C in the dark for 5 days, with the exception of *Pyiw*, which was incubated at 10°C for 7 days. The diameter of mycelial growth of isolates on MM and each carbon source was measured and the proportional growth rate relative to growth on V8 juice agar (diameter growth on agar with sole carbon source/growth on V8 juice agar ×100) was determined. The experiment was repeated twice.

### CAZy annotation

The whole genome shotgun projects of *Pythium* spp. are available in DDBJ/EMBL/GenBank under the accession numbers: AKXX00000000 for *Pyap*, AKXY00000000 for *Pyar*, AKXZ00000000 for *Pyir*, AKYA00000000 for *Pyiw*, ADOS00000000 for *Pyuu*, AKYB00000000 for *Pyus*, and AKYC00000000 for *Pyve*. The genome assemblies, transcript sequences, and protein sequences are also available for download and BLAST searching at *Pythium* Genome Database (PGD) website (http://pythium.plantbiology.msu.edu/, see download and BLAST pages) along with the annotation files in GFF3 format and the functional annotation of the gene models (http://pythium.plantbiology.msu.edu/download.shtml), which were used for CAZy annotation. The genome assembly and annotation files are also available for download from the Dryad Digital Repository (http://datadryad.org/) at this DOI (doi:10.5061/dryad.h748p). WGS and RNA-seq reads of *Pythium* spp. are available in the NCBI Short Read Archive (SRA) under the accessions SRP006957 and SRP006964, respectively. CAZy annotation of genomes other than *Pythium* spp. was conducted using the assembly 1 of the *Ph. infestans* genome released by Broad Institute (http://www.broadinstitute.org/annotation/genome/phytophthora_infestans/MultiHome.html), assembly 1.1 of the *Ph. sojae* and *Ph. ramorum* genomes released by the Joint Genome Institute (http://genome.jgi-psf.org/Physo1_1/Physo1_1.home.html; http://genome.jgipsf.org/ramorum1/ramorum1.home.html), and assembly V8.3.2 of the *H. arabidopsidis* from VBI Microbial Database V6.0 [94].

The CAZymes-encoding genes of *Pythium* spp., *Phytophthora* spp. and *Ha* were predicted automatically using CAT [40] and dbCAN [41], which are based on the CAZy (Carbohydrate-Active Enzyme) database classification [32]. Annotation was first carried out according to the two approaches available at CAT using the standard parameters, as described (Adhikari, *et al.* companion paper, PLoS One, this issue). Briefly, a bi-directional BlastP search of the protein-encoding ORFs from each genome [2], [10], [12], [13] was performed against the entire non-redundant sequences of the CAZy database. This was followed by annotation of the sequences using the PFAM domain database [95] and assignment of PFAM domains to the CAZy families. In this study, we also examined CAZymes using protein domains signatures search, via hidden Markov models constructed for each one of the CAZy families by dbCAN [41]. Both CAT and dbCAN results were combined and matches were considered positive when the E-value was less than 10^−05^. Positive hits were automatically annotated for a signal peptide using SignalP (http://www.cbs.dtu.dk/services/SignalP/) [96], GPI-anchor (http://gpi.unibe.ch/) [97] and transmembrane domain (http://phobius.binf.ku.dk/) [98]. InterPro families were assigned to the inferred CAZymes using InterProScan [99]. The CAZymes were categorized according to the type of reaction catalyzed: carbohydrate esterases (CE), glycoside hydrolases (GH), glycosyl transferases (GT), polysaccharide lyases (PL) and carbohydrate-binding modules (CBM), as described by Cantarel *et al.* [32]. The data generated by the analyses shown in Tables 1 and S1 were used in R [100] to produce box plots representing the central tendencies and distributions for each genus (Fig. S1). The values of zero were replaced by 0.1 in order to make log scale representation of the data possible. The pairwise differences between *Pythium* and *Phytophthora* (Fig. 3) were estimated with GLM in R, using loglinear/poisson model. The total number of genes per species (Adhikari, *et al.* companion paper, PLoS One, this issue) was used as centered covariate to normalize the data for the average number of genes. The *Pythium* effect was coded with dummy variables so that each coefficient represented the difference from *Phytophthora* for a given enzyme, providing significance value for each difference.

### Phylogenetic analyses

The nucleotide sequences corresponding to the 28S rRNA gene from the surveyed oomycetes and the predicted proteins encoding xyloglucan-β-1,4-D-endoglucanases (GH12), endoxylanases (GH10/GH11) and cutinases (CE5) were subjected to phylogenetic analyses. For the 28S (Fig. 1) and endoxylanase (Fig. 5) analyses, sequences of the diatoms *Thalassiosira pseudonana* (XP_002290930) [101] and *Phaeodactylum tricornutum* (XP_002178502) [102] were used as the outgroup, while GH12-sequence of *Aspergillus clavus* (XP_001269687) and cutinase sequence of *Frankia* sp. EUN1f (ZP_06415970) were used as outgroup in Fig. 4 and Fig. 7, respectively, since these genes are not present in the diatoms genomes. Sequences were aligned by ClustalW [103] and trimmed in Mega5 [104]. Phylogenetic analyses were performed using the MrBayes program for Bayesian analysis [105], using the general time-reversible model with inverse-gamma rates (nucleotide) or blosum (protein) of evolution for 300,000 generations. Phylogenetic trees were drawn and formatted in Mega5 [104]. Branches with bootstrap value less than 70 were collapsed. The phylogenetic tree of cutinase genes was displayed using the Interactive Tree of Life (iTOL) web server (http://itol.embl.de/) [106].

## Supporting Information

Figure S1 **Number of predicted carbohydrate-active enzymes (CAZymes) and the genome size of** ***Pythium*** **,** ***Phytophthora*** **and** ***Hyaloperonospora*** **.** This is based on the data from Table 1 (A) and Table S1 (B). The line in the middle of the box is the median, the diamond symbol is the average, the bottom and the top of the box are the 25th and 75th percentiles and the whiskers are 1.5 times the interquartile range above and below the box limits. The dots are outliers, *i.e.* beyond ±2.7 standard deviations.
(PDF)

Table S1 **Comparison of carbohydrate-degrading enzymes (CAZymes) encoded by Oomycota genomes using CAT and dbCAN.**
(DOCX)

Table S2 **Expressed genes of** ***Pythium*** **CAZyome.**
(XLSX)

Table S3 **Carbon sources.**
(XLSX)
