# Supplementary figures and images for: Carbohydrate-Active Enzymes in Pythium and Their Role in Plant Cell Wall and Storage Polysaccharide Degradation
Source: PLoS One. 2013 Sep 12;8(9):e72572. doi: 10.1371/journal.pone.0072572 (PMC3772060; doi:10.1371/journal.pone.0072572)

A

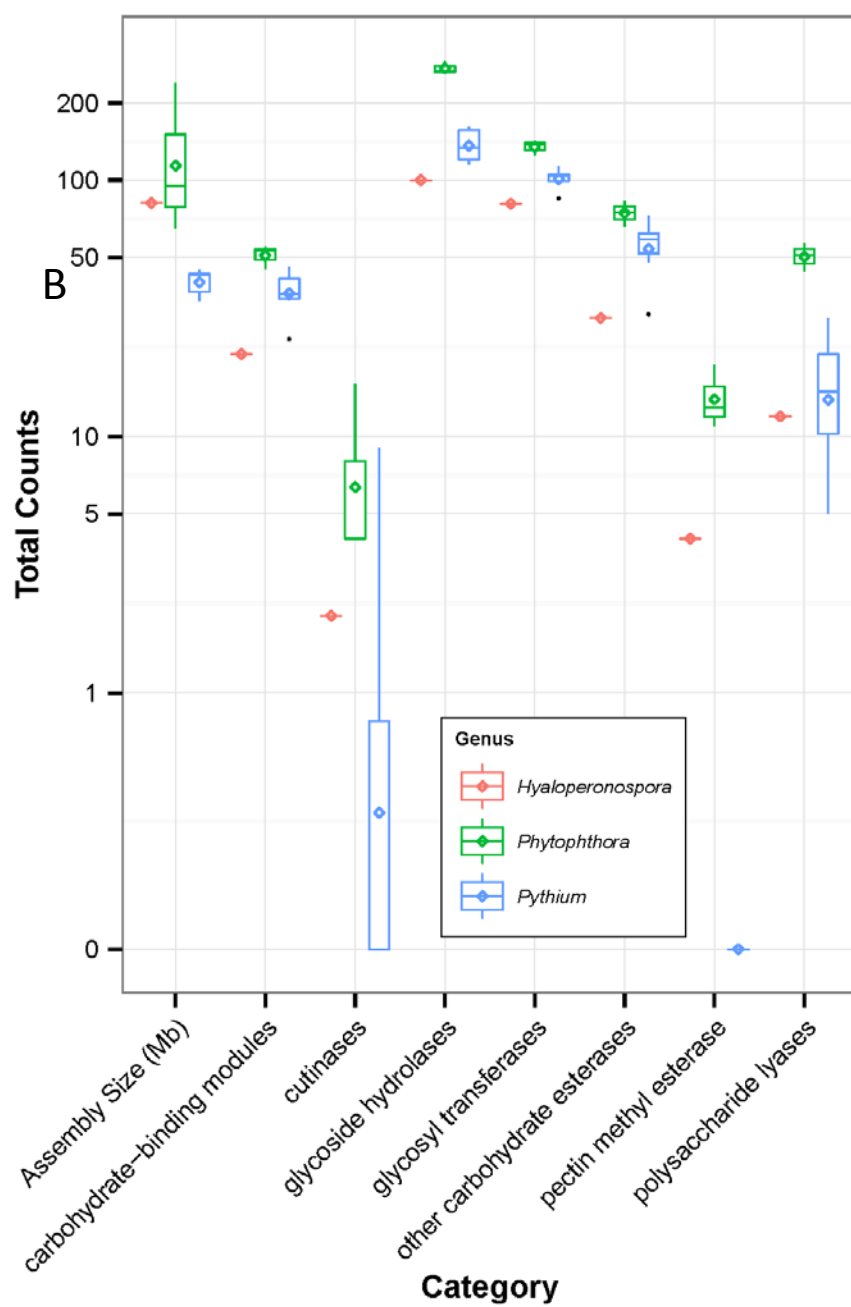

B

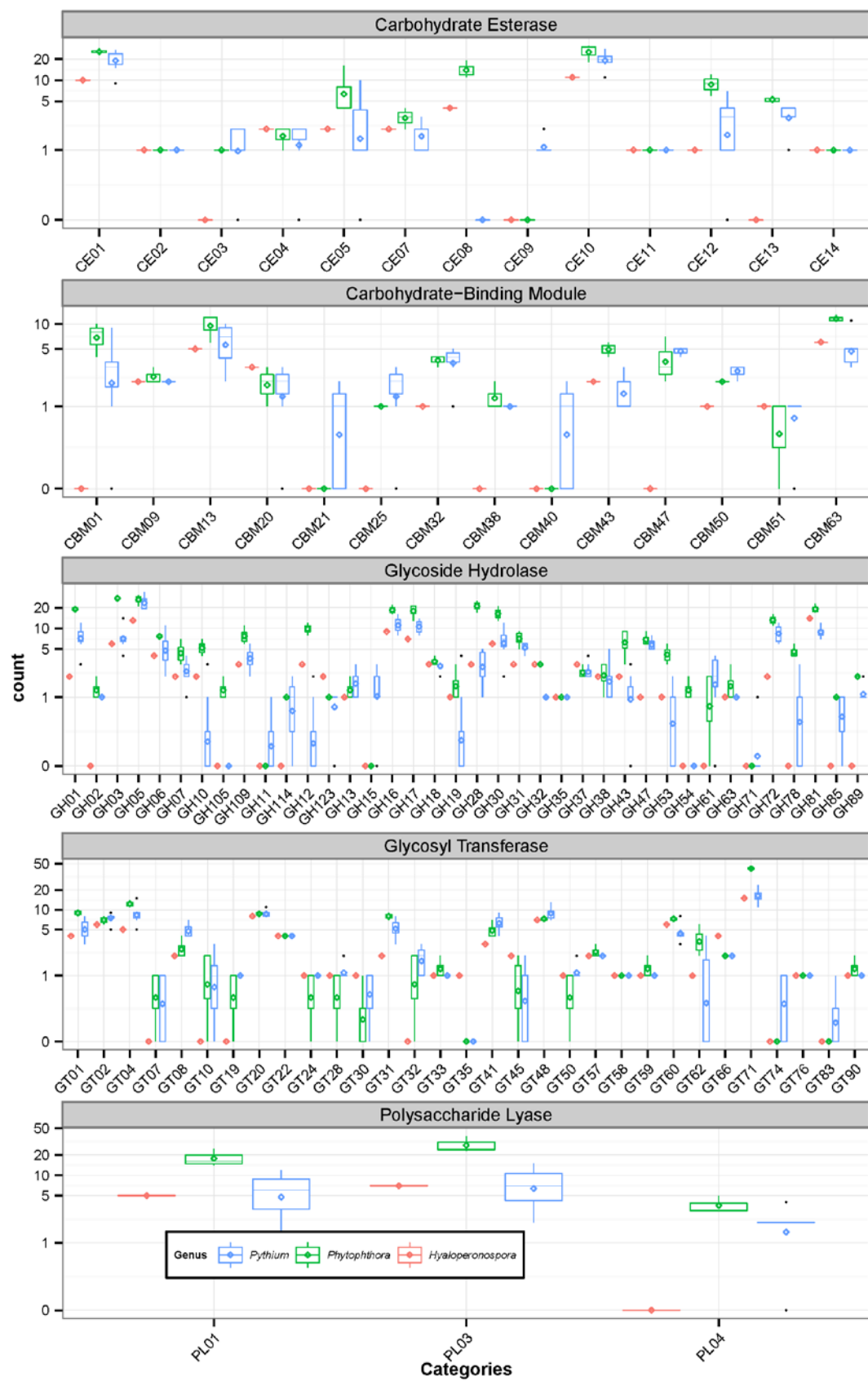

Supplement: Figure S1 — Number of predicted carbohydrate-active enzymes (CAZymes) and the genome size of Pythium , Phytophthora and Hyaloperonospora . This is based on the data from Table 1 (A) and Table S1 (B). The line in the middle of the box is the median, the diamond symbol is the average, the bottom and the top of the box are the 25th and 75th percentiles and the whiskers are 1.5 times the interquartile range above and below the box limits. The dots are outliers, i.e. beyond ±2.7 standard deviations. (PDF) [file pone.0072572.s001.pdf]
