# Supplementary material for: Carbohydrate-Active Enzymes in Pythium and Their Role in Plant Cell Wall and Storage Polysaccharide Degradation
Source: PLoS One. 2013 Sep 12;8(9):e72572. doi: 10.1371/journal.pone.0072572 (PMC3772060; doi:10.1371/journal.pone.0072572)
Supplement: Table S1 — Comparison of carbohydrate-degrading enzymes (CAZymes) encoded by Oomycota genomes using CAT and dbCAN. (DOCX) [file pone.0072572.s002.docx]

**Table S1.** Comparison of carbohydrate-degrading enzymes (CAZymes) encoded by Oomycota genomes using CAT and dbCAN.

|  | *Pyap* | *Pyar* | *Pyir* | *Pyiw* | *Pyuu* | *Pyus* | *Pyve* | *Phra* | *Phso* | *Phin* | *Ha* |
| --- | --- | --- | --- | --- | --- | --- | --- | --- | --- | --- | --- |
| CBM01 | 1 | 0 | 3 | 9 | 4 | 3 | 3 | 8 | 10 | 4 | 0 |
| CBM09 | 2 | 2 | 2 | 2 | 2 | 2 | 2 | 3 | 2 | 2 | 2 |
| CBM13 | 10 | 9 | 3 | 9 | 5 | 2 | 7 | 12 | 12 | 6 | 5 |
| CBM20 | 2 | 1 | 2 | 3 | 2 | 0 | 3 | 3 | 1 | 2 | 3 |
| CBM21 | 0 | 0 | 2 | 1 | 2 | 1 | 0 | 0 | 0 | 0 | 0 |
| CBM25 | 2 | 3 | 2 | 0 | 2 | 1 | 3 | 1 | 1 | 1 | 0 |
| CBM32 | 3 | 4 | 5 | 5 | 4 | 1 | 4 | 3 | 4 | 4 | 1 |
| CBM38 | 1 | 1 | 1 | 1 | 1 | 1 | 1 | 2 | 1 | 1 | 0 |
| CBM40 | 2 | 0 | 1 | 0 | 2 | 1 | 0 | 0 | 0 | 0 | 0 |
| CBM43 | 3 | 1 | 1 | 1 | 2 | 1 | 2 | 5 | 6 | 4 | 2 |
| CBM47 | 4 | 4 | 5 | 5 | 5 | 5 | 5 | 2 | 3 | 7 | 0 |
| CBM50 | 3 | 3 | 3 | 3 | 3 | 2 | 2 | 2 | 2 | 2 | 1 |
| CBM51 | 1 | 1 | 0 | 1 | 1 | 1 | 1 | 1 | 0 | 1 | 1 |
| CBM63 | 4 | 5 | 5 | 5 | 11 | 3 | 3 | 13 | 11 | 11 | 6 |
| Total | 38 | 34 | 35 | 45 | 46 | 24 | 36 | 55 | 53 | 45 | 21 |
|  |  |  |  |  |  |  |  |  |  |  |  |
|  | *Pyap* | *Pyar* | *Pyir* | *Pyiw* | *Pyuu* | *Pyus* | *Pyve* | *Phra* | *Phso* | *Phin* | *Ha* |
| CE01 | 27 | 24 | 23 | 15 | 24 | 9 | 19 | 27 | 24 | 26 | 10 |
| CE02 | 1 | 1 | 1 | 1 | 1 | 1 | 1 | 1 | 1 | 1 | 1 |
| CE03 | 2 | 2 | 0 | 1 | 1 | 1 | 2 | 1 | 1 | 1 | 0 |
| CE04 | 1 | 2 | 2 | 2 | 2 | 0 | 2 | 2 | 2 | 1 | 2 |
| CE05 | 10 | 7 | 1 | 2 | 1 | 1 | 0 | 4 | 16 | 4 | 2 |
| CE07 | 1 | 1 | 2 | 1 | 2 | 2 | 3 | 2 | 4 | 3 | 2 |
| CE08 | 0 | 0 | 0 | 0 | 0 | 0 | 0 | 13 | 19 | 11 | 4 |
| CE09 | 2 | 1 | 1 | 1 | 1 | 1 | 1 | 0 | 0 | 0 | 0 |
| CE10 | 17 | 28 | 23 | 19 | 21 | 11 | 20 | 29 | 31 | 18 | 11 |
| CE11 | 1 | 1 | 1 | 1 | 1 | 1 | 1 | 1 | 1 | 1 | 1 |
| CE12 | 4 | 7 | 1 | 0 | 4 | 1 | 3 | 6 | 12 | 9 | 1 |
| CE13 | 3 | 4 | 3 | 4 | 4 | 1 | 3 | 5 | 6 | 5 | 0 |
| CE14 | 1 | 1 | 1 | 1 | 1 | 1 | 1 | 1 | 1 | 1 | 1 |
| Total | 70 | 79 | 59 | 48 | 63 | 30 | 56 | 92 | 118 | 81 | 35 |
|  |  |  |  |  |  |  |  |  |  |  |  |
|  | *Pyap* | *Pyar* | *Pyir* | *Pyiw* | *Pyuu* | *Pyus* | *Pyve* | *Phra* | *Phso* | *Phin* | *Ha* |
| GH01 | 3 | 12 | 8 | 10 | 8 | 7 | 6 | 17 | 20 | 20 | 2 |
| GH02 | 1 | 1 | 1 | 1 | 1 | 1 | 1 | 1 | 2 | 1 | 0 |
| GH03 | 7 | 7 | 8 | 6 | 7 | 4 | 14 | 28 | 29 | 25 | 6 |
| GH05 | 19 | 30 | 24 | 20 | 34 | 22 | 19 | 30 | 28 | 21 | 13 |
| GH06 | 2 | 11 | 7 | 4 | 6 | 5 | 3 | 8 | 8 | 7 | 4 |
| GH07 | 3 | 4 | 3 | 3 | 1 | 2 | 2 | 4 | 7 | 3 | 2 |
| GH10 | 0 | 3 | 0 | 0 | 0 | 0 | 1 | 7 | 5 | 4 | 2 |
| GH11 | 1 | 1 | 0 | 0 | 0 | 0 | 0 | 0 | 0 | 0 | 0 |
| GH12 | 0 | 2 | 0 | 0 | 0 | 0 | 1 | 8 | 12 | 10 | 3 |
| GH13 | 1 | 1 | 3 | 2 | 2 | 2 | 1 | 2 | 1 | 1 | 1 |
| GH15 | 2 | 1 | 1 | 1 | 3 | 2 | 0 | 0 | 0 | 0 | 0 |
| GH16 | 8 | 10 | 9 | 10 | 16 | 12 | 15 | 16 | 18 | 22 | 9 |
| GH17 | 11 | 14 | 10 | 8 | 13 | 8 | 12 | 21 | 21 | 13 | 7 |
| GH18 | 3 | 3 | 3 | 3 | 3 | 2 | 3 | 3 | 4 | 3 | 3 |
| GH19 | 1 | 0 | 0 | 0 | 0 | 0 | 4 | 1 | 1 | 3 | 1 |
| GH28 | 5 | 3 | 2 | 1 | 5 | 2 | 4 | 17 | 25 | 22 | 3 |
| GH30 | 2 | 7 | 7 | 6 | 9 | 5 | 12 | 13 | 16 | 21 | 6 |
| GH31 | 6 | 4 | 5 | 6 | 5 | 5 | 6 | 5 | 8 | 9 | 3 |
| GH32 | 1 | 1 | 1 | 1 | 1 | 1 | 1 | 3 | 3 | 3 | 3 |
| GH35 | 1 | 1 | 1 | 1 | 1 | 1 | 1 | 1 | 1 | 1 | 1 |
| GH37 | 2 | 2 | 2 | 2 | 3 | 4 | 2 | 2 | 2 | 3 | 3 |
| GH38 | 1 | 2 | 2 | 2 | 1 | 1 | 5 | 3 | 3 | 1 | 2 |
| GH43 | 1 | 1 | 1 | 0 | 2 | 1 | 3 | 9 | 9 | 3 | 2 |
| GH47 | 6 | 8 | 5 | 5 | 5 | 7 | 5 | 9 | 6 | 6 | 1 |
| GH53 | 0 | 1 | 0 | 0 | 2 | 1 | 1 | 6 | 4 | 3 | 1 |
| GH54 | 0 | 0 | 0 | 0 | 0 | 0 | 0 | 1 | 2 | 1 | 0 |
| GH61 | 4 | 3 | 4 | 2 | 2 | 0 | 1 | 2 | 2 | 0 | 0 |
| GH63 | 1 | 1 | 1 | 1 | 1 | 1 | 1 | 3 | 1 | 1 | 1 |
| GH71 | 0 | 0 | 0 | 0 | 0 | 0 | 1 | 0 | 0 | 0 | 0 |
| GH72 | 7 | 6 | 10 | 12 | 11 | 9 | 6 | 11 | 16 | 13 | 2 |
| GH78 | 1 | 0 | 1 | 1 | 0 | 0 | 3 | 4 | 4 | 6 | 0 |
| GH81 | 9 | 9 | 8 | 10 | 8 | 7 | 12 | 18 | 23 | 17 | 14 |
| GH85 | 0 | 0 | 1 | 1 | 1 | 1 | 1 | 1 | 1 | 1 | 0 |
| GH89 | 1 | 1 | 1 | 1 | 2 | 1 | 1 | 2 | 2 | 2 | 0 |
| GH105 | 0 | 0 | 0 | 0 | 0 | 0 | 0 | 1 | 1 | 2 | 0 |
| GH109 | 3 | 4 | 4 | 3 | 6 | 2 | 5 | 7 | 6 | 11 | 3 |
| GH114 | 1 | 2 | 0 | 0 | 1 | 1 | 2 | 1 | 1 | 1 | 0 |
| GH123 | 1 | 1 | 1 | 1 | 1 | 0 | 1 | 1 | 1 | 1 | 2 |
| Total | 115 | 157 | 134 | 124 | 161 | 117 | 156 | 266 | 293 | 261 | 100 |
|  |  |  |  |  |  |  |  |  |  |  |  |
|  | *Pyap* | *Pyar* | *Pyir* | *Pyiw* | *Pyuu* | *Pyus* | *Pyve* | *Phra* | *Phso* | *Phin* | *Ha* |
| GT01 | 4 | 7 | 8 | 6 | 4 | 5 | 3 | 8 | 10 | 9 | 4 |
| GT02 | 9 | 8 | 8 | 7 | 8 | 5 | 8 | 7 | 6 | 8 | 6 |
| GT04 | 8 | 7 | 9 | 9 | 8 | 5 | 15 | 11 | 14 | 12 | 5 |
| GT07 | 0 | 0 | 1 | 1 | 1 | 1 | 0 | 0 | 1 | 1 | 0 |
| GT08 | 6 | 4 | 5 | 4 | 4 | 4 | 7 | 2 | 2 | 4 | 2 |
| GT10 | 0 | 2 | 3 | 1 | 0 | 1 | 1 | 2 | 0 | 2 | 0 |
| GT19 | 1 | 1 | 1 | 1 | 1 | 1 | 1 | 0 | 1 | 1 | 0 |
| GT20 | 8 | 11 | 8 | 8 | 9 | 9 | 8 | 8 | 9 | 9 | 8 |
| GT22 | 4 | 4 | 4 | 4 | 4 | 4 | 4 | 4 | 4 | 4 | 4 |
| GT24 | 1 | 1 | 1 | 1 | 1 | 1 | 1 | 1 | 0 | 1 | 1 |
| GT28 | 1 | 1 | 1 | 2 | 1 | 1 | 1 | 0 | 1 | 1 | 1 |
| GT30 | 1 | 0 | 1 | 1 | 1 | 1 | 0 | 0 | 0 | 1 | 1 |
| GT31 | 8 | 8 | 5 | 5 | 5 | 3 | 4 | 7 | 9 | 8 | 2 |
| GT32 | 3 | 2 | 2 | 3 | 1 | 1 | 1 | 2 | 2 | 0 | 0 |
| GT33 | 1 | 1 | 1 | 1 | 1 | 1 | 1 | 1 | 1 | 2 | 1 |
| GT35 | 0 | 0 | 0 | 0 | 0 | 0 | 0 | 0 | 0 | 0 | 1 |
| GT41 | 7 | 8 | 6 | 9 | 5 | 6 | 4 | 4 | 4 | 7 | 3 |
| GT45 | 0 | 0 | 1 | 1 | 2 | 1 | 0 | 0 | 2 | 1 | 2 |
| GT48 | 7 | 13 | 9 | 10 | 7 | 9 | 8 | 7 | 8 | 7 | 7 |
| GT50 | 1 | 1 | 1 | 2 | 1 | 1 | 1 | 1 | 0 | 1 | 1 |
| GT57 | 2 | 2 | 2 | 2 | 2 | 2 | 2 | 3 | 2 | 2 | 2 |
| GT58 | 1 | 1 | 1 | 1 | 1 | 1 | 1 | 1 | 1 | 1 | 1 |
| GT59 | 1 | 1 | 1 | 1 | 1 | 1 | 1 | 1 | 1 | 2 | 1 |
| GT60 | 5 | 8 | 4 | 4 | 4 | 4 | 3 | 8 | 7 | 7 | 6 |
| GT62 | 3 | 4 | 0 | 0 | 0 | 0 | 1 | 3 | 6 | 2 | 1 |
| GT66 | 2 | 2 | 2 | 2 | 2 | 2 | 2 | 2 | 2 | 2 | 4 |
| GT71 | 18 | 14 | 15 | 16 | 18 | 11 | 24 | 40 | 43 | 44 | 15 |
| GT74 | 1 | 0 | 0 | 1 | 1 | 1 | 0 | 0 | 0 | 0 | 0 |
| GT76 | 1 | 1 | 1 | 1 | 1 | 1 | 1 | 1 | 1 | 1 | 1 |
| GT83 | 0 | 0 | 0 | 0 | 1 | 1 | 0 | 0 | 0 | 0 | 0 |
| GT90 | 1 | 1 | 1 | 1 | 1 | 1 | 1 | 1 | 1 | 2 | 1 |
| Total | 105 | 113 | 102 | 105 | 96 | 85 | 104 | 125 | 138 | 142 | 81 |
|  |  |  |  |  |  |  |  |  |  |  |  |
|  | *Pyap* | *Pyar* | *Pyir* | *Pyiw* | *Pyuu* | *Pyus* | *Pyve* | *Phra* | *Phso* | *Phin* | *Ha* |
| PL01 | 7 | 1 | 6 | 2 | 12 | 5 | 11 | 14 | 25 | 16 | 5 |
| PL03 | 14 | 2 | 7 | 3 | 15 | 8 | 6 | 25 | 23 | 38 | 7 |
| PL04 | 0 | 2 | 2 | 2 | 2 | 2 | 4 | 5 | 3 | 3 | 0 |
| Total | 21 | 5 | 15 | 7 | 29 | 15 | 21 | 44 | 51 | 57 | 12 |

*Pythium aphanidermatum* (*Pyap*); *Py. arrhenomanes* (*Pyar*); *Py. irregulare* (*Pyir*); *Py. iwayamai* (*Pyiw*); *Py*. *ultimum* var. *ultimum* (*Pyuu*); *Py. ultimum* var. *sporangiferum* (*Pyus*); *Py.* *vexans* (*Pyve*); *Phytophthora ramorum* (*Phra*); *Ph. sojae* (*Phso*); *Ph. infestans* (*Phin*); and *Hyaloperonospora arabidopsidis* (*Ha*).
